# Exploring the dynamics between homelessness and healthcare access for Aboriginal and Torres Strait Islander women with traumatic brain injury from family violence: a qualitative study

**DOI:** 10.1186/s12889-026-26458-6

**Published:** 2026-05-08

**Authors:** Michelle Fitts, Karen Soldatic

**Affiliations:** 1 Institute for Culture and Society, Western Sydney University, Parramatta, NSW Australia; 2 Menzies School of Health Research, Charles Darwin University, Alice Springs, NT Australia; 3 Australian Institute of Tropical Health and Medicine, James Cook University, Townsville, QLD Australia; 4 Canada Excellence Research Chair, Toronto Metropolitan University, Toronto, ON Canada

**Keywords:** Aboriginal and Torres Strait Islander, Housing, Healthcare, Women, Family violence, Traumatic brain injury

## Abstract

**Background:**

Homelessness is a significant issue in the context of violence, particularly for Indigenous women in settler colonial countries, as are the long-lasting impacts of violence such as traumatic brain injury (TBI) including concussion. Understanding of the relationship between homelessness and healthcare access for TBI from violence among Indigenous women is critical for informing service delivery; however, research in this area remains limited. Situated within the broader experiences of accessing healthcare following violence-related TBI, this study aimed to explore the relational dynamics between violence, homelessness, healthcare access, and the implications for long term recovery and wellbeing.

**Methods:**

Using purposive and snowball sampling, semi-structured interviews and focus groups were completed with 18 Indigenous women who have experienced TBI from violence, 28 community members, and 90 community-based frontline workers to gather insights into the experiences of living with TBI family violence or supporting someone with this injury. All data were transcribed verbatim and analyzed using thematic analysis.

**Results:**

Two themes were identified regarding how responses to family violence-related homelessness created barriers for Indigenous women to access healthcare for TBI: (1) Housing service barriers affect access to healthcare and TBI management, and (2) The (in)visibility of TBI within crisis accommodation and housing services. The dominant experience for Indigenous women who had experienced violence and homelessness was characterized by complexity, uncertainty, and distress, largely due to service delays and barriers encountered across housing pathways. Some Indigenous women were required to relocate away from their home region to secure temporary accommodation. While crisis accommodation services were widely viewed as places of safety, many did not consider TBI in environmental design or service delivery. Multi-agency case management and outreach were identified as valuable approaches for improving healthcare access.

**Conclusions:**

The findings illustrate the importance of TBI-informed policy and practice within housing and homelessness services, especially for Indigenous women in rural and remote regions, alongside strengthened workforce training. Stronger linkages between women’s shelters, housing services, and healthcare systems - including concussion clinics - are critical for supporting both immediate and long-term care. Needs-based funding is required to ensure regional and remote housing systems can support women-led responses, including more streamlined transitions from crisis or short-term accommodation to secure housing.

## Introduction

In Australia, Aboriginal and Torres Strait Islander (hereafter respectfully, Indigenous) women and children are disproportionately affected by both homelessness and violence, with violence being the leading cause of homelessness for Indigenous women [1]. Emergency crisis shelters are the most-recognized forms of accommodation available to women experiencing family violence and their establishment were a cornerstone of the 1970s women’s movement [2]. However, in regional and remote landscapes, access is severely limited due to underfunding, resource constraits, and privacy concerns in small communities. 

 The shortage of safe crisis accommodation has profound consequences, affecting long-term accommodation trajectories for Indigenous women as well as their ability to access healthcare to stabilise their health and wellbeing to enable a full recovery [3]. It has become well established within the research in partnership with Indigenous communities across settler-colonial contexts, that the ongoing denial of safe crises accommodation and domestic violence services in rural and remote communities is associated with further violence. Indigenous women and their children have had to leave shelters without housing in place or have had to relocate outside of their home region to secure precarious accommodation (e.g., motels) that are unsafe and highly unsuited to women and children seeking a family violence response [4]. Timely, accessible and safe housing is therefore a major need of women and their children impacted by family violence that is not being met in regional and remote Australia [5].

This paper draws upon a large national project that interviewed Indigenous women who had experienced family violence alongside healthcare and service providers working in the area of family violence across two regional sites in Australia [6, 7]. The aim of the project was to understand the ways in which hospitals and community-based services provide care, support and treatment to Indigenous women and their children in rural and remote regions immediately post a family violence event and, the impact of such services upon the long-term health and wellbeing of Indigenous women. The focus of the study was on Indigenous women who had experienced a traumatic brain injury (TBI) as a result of family violence and the capacity of the system to respond effectively and responsively to their unique injury and its impact. This paper provides some broader context of the regions in which the study was undertaken to demonstrate the severe constraints that Indigenous women experience and its impact upon their long-term recovery as women, mothers, and members of their communities.

### Context of the study: the pathway from violence to homelessness for Indigenous women in regional and remote Australia

The study sites were in the Northern Territory and Queensland, where Indigenous women and children experience the highest rates of homelessness and overcrowding nationally [8]. Inadequate housing has a bi-fold effect. First, it has been identified as one of the primary reasons for the continuity of family violence as Indigenous women are unable to find alternative housing for themselves and their children [9, 10]. Secondly, homelessness can also impede the likelihood of reunification between Indigenous mothers and their children occurring within a short time frame as local authorities will remove children from their mothers if there is nowhere safe for them to return to [10]. In regional and remote communities, these issues are exacerbated by the fact that there is almost no local emergency housing in small, under-serviced Indigenous communities, where residents are often closely connected.

Although a substantial body of research has documented the association between family violence and housing in Australia, relatively little is known about Indigenous women’s experiences accessing healthcare in response to family violence resulting in TBI and the impact of these different housing journeys [11, 12]. Thus, while there is a strong association between homelessness and family violence, more targeted research is needed to understand how Indigenous women who are experiencing homelessness can be supported by housing and crisis accommodation services to access healthcare for TBI, both immediately after the event and in the long term. This is particularly critical as broader structural issues around historical disadvantage, discriminatory health systems and severe levels of poverty generated by settler colonial dispossession, means that Indigenous women and their children are uniquely susceptible to poor outcomes over the longer term. Settler colonialism has disrupted Indigenous housing, family and kinship relations of care by displacing Indigenous peoples from their ancestral lands, separating children from their families and implementing exclusionary socio-spatial structures all of which continue to undermine Indigenous women’s access to services [13, 14]. Thus, any policy innovations to shore up resources for effective, sustainable long term health system responses for Indigenous women, need to account for the significant complexity of rural and remote healthcare in settler colonial contexts.

### Traumatic brain injury - the invisible disability generated by physical violence

For many women who survive violence, the impact of physical violence lasts long after the abuse ends [15]. Violent behaviours including a direct blow to the head, being hit in the head hit with a fist or hard object; a force transmitted from the body to the head, shaking of the body or the head, or by external pressure that causes the blood vessels and/or air passages to close depriving the brain of oxygen can result in a range of injuries including TBI [16–19]. Cases of TBI can impart significant and long-lasting psychological, behavioural and emotional symptoms [20]. For example, once considered a mild injury, there is mounting recognition that concussion affects major life functions of a person, such as reading, sleeping and concentration and is a significant disability [21].

TBI can occur even when a loss of consciousness is not experienced and when there are no signs of injury visible (e.g., wound, bruising, blood). Yet, for women, including Indigenous women, these aspects of the injury as well as a lack of awareness of the lasting impacts of violence on the brain, and fear of further violence all contribute towards women not accessing healthcare immediately following the injury [22, 23]. Structural racism including fear of child removal by child protection services is a further reason Indigenous women decide not to access healthcare following TBI [24]. TBI can also go unrecognised or undiagnosed by frontline professionals because of the lack of knowledge of the association between family violence and TBI as well as lack of screening protocols within frontline service assessment and practice [22, 25–27]. Women have reported cognitive deficits such as memory loss, confusion, difficulty following directions and difficulty concentrating [27, 28]. Women who access services such as crisis accommodation are likely to experience multiple head injuries from family violence over a prolonged period of time with research suggesting that an accumulation of injuries can result in more severe symptoms [20, 28, 29]. As repeated physical abuse can accelerate the development of neurodegeneration and can result in the brain degeneration of chronic traumatic encephalopathy [30], crisis accommodation and housing services play a critical role in supporting Indigenous women to access healthcare. Timely access to healthcare following TBI is shown to improve recovery outcomes. This is critically important, as Indigenous women who are experiencing homelessness are more likely to have poor health outcomes and be high users of acute health services [31].

### A note on terminology

It is important to note that while violence is defined differently across cultures, in this study the term family violence is used as it is often the preferred term in relation to framing family violence in the Indigenous context in Australia [32]. Unlike other terms, family violence reflects a holistic understanding that encompasses violence occurring within families and communities and, importantly, recognises broader aspects of the phenomenon, including elder abuse, family feuding, and child abuse [32]. In Australia, there is not a clear, single definition of homelessness. The Australian Bureau of Statistics (ABS) defines being homeless as when a person lacks one of more elements that represent a home and captures a range of different circumstances. For example, when a person’s living arrangement is a dwelling that is inadequate, when the person has no tenure or, if their initial tenure is short and not extendable, or when the arrangement does not allow the person to have control of and nor access to space for social connections [33]. Another definition used is when a person is living in non-conventional accommodation or has no shelter (such as, living on the street, living in a caravan, cabin or tent, living in a caravan park, or in a motor vehicle), or short-term or emergency accommodation [34].

## Methods

### Ethics approval

This study aimed to assess how being homeless or unstably housed affects access to healthcare for TBI treatment and support among Indigenous women living in regional and remote Australia. This study was approved by the Central Australian Human Research Ethics Committee (CA-21-4160), Western Sydney University Human Research Ethics Committee (H14646) and Townsville Hospital and Health Service Human Research Ethics Committee (HREC/QTHS/85271). Research approvals were also received from Aboriginal community-controlled, legal and family violence research committees and boards. Guidelines for completing research with Indigenous peoples in Australia were followed at each of the project locations [35]. All participation was voluntary. All participants signed consent forms.

### Positionality statement

The researchers are non-Indigenous academics whose research has a particular focus on women from Indigenous and minority backgrounds who experience barriers to service access, support in addition to effective care and treatment following violence. They also have experience in disability policy, alcohol and injury research with a focus on health equity and accessibility within remote healthcare. Given this prior research, the researchers spent considerable time in thinking through potential modifications that would be required for each of the field sites and worked extensively with community members to shape and frame the research praxis overall [36]. The researchers were guided by community members, Elders and service providers within each site. Feedback was integrated into each round of interviews. As described elsewhere [7], this enabled the researchers to build a process that was consistently guided by local community members and participants to ensure that processes were responsive to local community requirements from initial design, implementation, analysis and rigorous practices of member checking to ensure that data interpretation, findings and presentation was reflective of localised interpretative understandings and experiences. This rigorously grounded practice was designed to build upon local Indigenous protocols in guiding non-Indigenous researchers working alongside community.

### Participants

Two project locations were part of the study, one regional region in Queensland and one remote region in the Northern Territory. To protect the identity of communities involved in the project, the names of the two regions are not provided. This study draws upon interviews and focus groups completed with Indigenous women with lived experience of TBI from family violence, community-based groups (e.g., women’s groups and Elders’ groups) and frontline workers from community-based services. The project inclusion criteria were broad to ensure Indigenous women who have experienced any severity level of TBI could take part in the study. In order to generate a comprehensive understanding of the dynamics between homelessness, family violence and healthcare access, the research team contacted women’s groups, Elders groups as well as community-based frontline workers in both medical (e.g., nurses, general practitioners, and specialists) and non-medical professions (e.g., housing and crisis accommodation, and social, welfare, disability, family violence, and community legal services).

### Participant recruitment

A multipronged recruitment strategy involving purposeful and snowball sampling was applied to recruit participants to the study. To begin, all staff from community-based services were invited to participate in a face-to-face interview or discussion group with a member of the research team. Team leaders at community-based services were emailed copies of the participant information sheet and consent form and a summary of the project and contact details of the lead researcher. Staff interested in participating in the study contacted a researcher via telephone or email. Once contact had been made, the researcher liaised with each potential participant to find a suitable time to meet in person, to discuss the information disseminated in the project, and, if consent was given, to conduct an interview or focus group. Recruitment from community-based services also involved attendance at staff meetings (face-to-face and online) to present information on the project and advise avenues of staff participation if interested. Snowball sampling was also used, with participants asked to recommend other relevant staff to participate in the study [37]. Some staff were recommended to the research team and therefore contacted directly. Community-based service interviews, dyadic interviews and focus groups took place at the service office. Almost all frontline professionals were women (85 participants; 94%). About half of frontline professionals identified as Aboriginal and/or Torres Strait Islander (40 participants, 44%).

Following interviews with community-based services, Indigenous women were recruited through the support of community-based organisations. The community-based organisations approached had both consistent contact with Indigenous women living with TBI from family violence including Aboriginal community-controlled and mainstream legal and health services. TBIs were confirmed through staff who had access to the women’s medical records, legal records, or other social service records. Staff from community-based organisations approached Indigenous women clients who met the inclusion criteria about participation in the project. At this initial conversation about the project, the staff member explained the project supported by a short video about the project and/or project flipchart. The project commissioned Indigenous women artists to complete pieces of artwork to include in the flipchart to create opportunity to safely explain and discuss the project. Indigenous women who were interested in hearing more about the project and possibly participating in the project met with one or two members of the research team. Initial meetings usually took place at the service office who made the referral (e.g., primary healthcare clinic), at their accommodation (e.g., hostel, womens shelter) or the women’s group’s meeting space.

### Data collection

Prior to data collection, in most cases TBI education sessions were delivered to women groups and community-based services to ensure all potential participants had the same understanding of the brain, how TBI affects brain function and how to support someone who has experienced TBI [7]. This approach was also influenced by Indigenist methodology, which emphasis the importance of relationality, reciprocity and respect. Data collection in this study occurring between January 2022 and December 2023. A flexible interview guide was used to focus the conversation on the care, support and treatment Indigenous women head injuries from family violence; factors that enhanced the healthcare and interactions Indigenous women had at the hospital; perceptions of cultural, social and structural barriers to healthcare access, and policies and practices that could be implemented to enhance the healthcare Indigenous women receive in the hospital and post-discharge [38]. The interview schedule and project flipchart outlined opening comments about the topic of TBI, introductory questions to create space for there to be information shared between the research team members and participants to build rapport and relationships as well as create space for Indigenous women to discuss and explore their experiences of family violence, TBI and service system access. While a topic list guided the process, Indigenous women were encouraged to talk freely, telling their stories in their own way. The flexible interview guide allowed exploration of additional issues raised by Indigenous women within the initial interview and in subsequent interviews. Indigenous women with lived experience of TBI from violence were aged between 26 and 68 years of age at the time of the interview. The average age was 40 years.

### Data management and analysis

Ninety community-based service provider staff, from across the two regions participated in an interview or focus group for the project. Individual interviews, dyadic interviews and focus groups were digitally recorded and transcribed verbatim by a professional transcription service. Each transcript was reviewed for accuracy. Written notes from each interview and focus group were also typed up in a word document and allocated a participant number. The transcripts were managed using Nvivo software. Thematic analysis was used as there is limited evidence on the perspectives of Indigenous women living with TBI from family violence [39]. Implementing this approach to analysis allows themes to emerge from patterns of meaning and unexpected perceptions [40]. Transcripts were read repeatedly by the authors to familiarize themselves with the data. Initial codes were identified, and the findings related to these codes summarized. Initial codes either related specifically to the interview topics or were derived from the data. The data were organised into themes based on patterns identified, with further review of themes and their relationships to each other. The initial codes and data coded were discussed between both authors and further refined. The results were presented to members of the project advisory group as well as Elders from the two project locations at intervals throughout the analysis, to confirm interpretation and further elaborate issues.

### Findings

Homelessness and housing insecurity were identified by participants as affecting how and when Indigenous women engage with acute care and community-based primary healthcare following TBI. Two themes emerged from experiences of Indigenous women living with TBI from family violence: (1) Housing service barriers affect access to healthcare and TBI management, and (2) The (in)visibility of traumatic brain injury within crisis accommodation and housing. Each theme was informed by the voices of Indigenous women from both regions.

### Housing service barriers affect access to healthcare and TBI management

#### How housing blockages make a pathway a repetitive cycle

The housing status of Indigenous women who had experienced violence-related TBI was dominated by prolonged periods of living with family members, often in overcrowded homes as well as accessing homelessness services, living in hostels and living in public spaces (e.g., parks). A major issue described by all participant groups was the limited housing options immediately available to Indigenous women leaving violence: *No safe housing once they leave.* (Womens Group, region 1) At times, Indigenous women were reliant upon staying with family members: *They move around*,* can live at aunties*,* grandmothers place but they can move around and go from home to home* (Womens Group, region 1). For Indigenous mothers, like Donna, crisis accommodation services were unable to support the housing needs of some women who had the care responsibilities of children:

*It’s just a circle*,* go backwards. They [partner] say they’ll change. You feel sorry for them*,* but he never do. Then you would circle back to the women’s shelter*,* staying with family. Womens shelter*,* hospital*,* I can get a good rest there. When you have children*,* you have to go back to your home. I have four boys and two girls*,* there is no women’s shelter that can take us.* (Donna, region 2)

Another barrier to accessing crisis accommodation arose for Indigenous women caring for older male children, as many women’s shelters had policies that only permitted male children up to 12 years of age to stay at the service. As a result, some women were unable to access crisis accommodation as doing so would require separating from their children. 

Crisis accommodation was described as a vital service that provides respite and safety, allowing women time to make decisions about their healthcare while establishing connections with necessary support services. However, due to existing service barriers, these critical moments for healthcare decision-making were often unavailable to Indigenous women in regional and remote areas. Access to service supports post-presentation to hospital varied depending on the availability of crisis accommodation. While some Indigenous women were referred to or transported to crisis accommodation directly from the local hospital, other Indigenous women - particularly those in region 2 - reported that these services were frequently at capacity. Without access to crisis accommodation, some Indigenous women described being discharged from hospital back into homelessness, with limited direct contact with community-based support services:

*I didn’t always go hospital. Sometimes you can’t go*,* you get held back. Then you go. Nothing from the hospital*,* once you leave*,* you can’t contact them again. You call the main number and they told me to contact my local doctor. Why did I go? No one followed-up with me.* (Lila, region 2)

One crisis accommodation service had introduced a small fee to help cover rising service delivery costs that could not be met due to limited funding. Barriers were also reported across other parts of the housing pathway by all participant groups. In both study regions, frontline professionals from community-based organisations described the public housing system as being in *‘crisis’*, with a reported 10-year wait list in region 1 and described as a *‘massive issue’* in region 2.

#### Alternative housing solutions are not fit for purpose

Alternative accommodation options offered to Indigenous women by family violence services included unsafe options such as tents. Use of short-term accommodation options outside of the local region, such as motels, were viewed as problematic as Indigenous women and their children were required to relocate from social and family networks where they received informal supports during this difficult period while simultaneously, attempting to navigate new healthcare services where they often did not have an existing relationship with staff and services. There were also concerns that relocation added new transport costs for Indigenous women, who often did not have access to a working, registered vehicle nor hold current valid driver licence. Being housed in accommodation that was not fit for purpose, such as motels, was viewed by frontline services as further compounding the risks for further or new harms for Indigenous women and their children.

Aboriginal hostels were another form of accommodation accessed by Indigenous women in some instances; however, these options were temporary and often unsuitable for women with children. In the examples shared, Indigenous women were staying with their children in single-bedroom accommodation, resulting in severe overcrowding for many women with children. Hostel rooms lacked adequate space for children's belongings, such as clothing and toys, and facilities like fridges were frequently too small to store all items for large families. Despite these limitations, some aspects of Aboriginal hostels were considered supportive for Indigenous women, including the provision of meals, opportunities to connect with extended family members and community, and access to transport. Nevertheless, living in temporary accommodation was invariably described as challenging, with Indigenous women drawing attention to not having enough space for themselves and their children. As hostels and motels were not designed to accommodate the complexities associated with TBI, Indigenous women reported difficulties implementing care regimes from information provided by healthcare professionals. Many also struggled with the absence of quiet spaces to rest and manage symptoms such as fatigue and sensitivity to noise. As Josie explains, the lack of appropriate accommodation contributed to her contemplating relocation outside of her home region so that she and her children could gain greater access to services in a major city:

*I live here with three of my four boys*,* it is a one bedroom place. We have been here months. I am ready to go to [name of city] to get better opportunities for my family*,* we can’t stay here. I want to have a front yard for my boys to play around*,* to have toys.* (Josie, region 1)

### The (in)visibility of traumatic brain injury within crisis accommodation and housing

#### Missed opportunities to discuss traumatic brain injury

Indigenous women with lived experience of violence-related TBI, as well as members from Indigenous women's groups, reflected on the ways the housing service sector had transformed its responses to family violence within their communities and broader region. Since the establishment of crisis accommodation services in the 1970s, these spaces have increasingly become places where Indigenous women experiencing family violence can access a range of supports, including healthcare. Participants also described broader community conversations about the harms and impacts of family violence emerging through locally-led and national campaigns aimed at ending violence. While these developments were recognized as important progress, Indigenous women with lived experience and womens groups noted that awareness and understanding of TBI resulting from family violence was still relatively new within the community. As Marlee explained, unlike other injuries - such as non-fatal strangulation - TBI had not been something she had previously been asked about when accessing crisis accommodation services:

*No one has spoken to me about the effects of my assaults on my brain. I have been asked about being chocked; about injuries you can see. I have these lumps and bumps around here and on top (Marlee runs her fingers over forehead and around the sides of her head). They sometimes ache. No one at the shelter asked me about brain injury.* (Marlee, region 2)

Marlee's reflections were echoed by many Indigenous women and women's groups involved in this study, who reported that while awareness of non-fatal strangulation had increased in recent years, awareness of the connection between physical violence to the head and harm to the brain remained limited.

#### Unrealistic expectations placed upon women by housing services

During times of crisis and the forced reliance on temporary accommodation, Indigenous women described feeling overwhelmed by the expectations placed on them by some service systems, given the turmoil of their circumstances and the very real risk of impending homelessness. Women were often required to remember and attend multiple appointments, follow instructions from healthcare providers, and take immediate steps to access support services. While these requirements were often essential, they were coupled with the urgent need to gather and maintain important documentation in order to secure safety for themselves and their children. This was particularly challenging because, when fleeing violence, women rarely had the opportunity to collect basic identity documents - such as healthcare cards, social security information, banking details, and other personal records. Leaving a violent partner or family member often occurs rapidly, making it difficult to secure the documentation required to navigate service systems and access support.

Significantly, for women experiencing violence-related TBI, these commonly reported challenges were further compounded by the effects of the injury itself. Indigenous women with lived experience described severe difficulties with retaining critical personal information, as well as changes in concentration and attention - particularly in high-stress situations - alongside cognitive and emotional fatigue. These impacts made navigating complex housing and healthcare systems especially difficult. As a result, Indigenous women often felt isolated when attempting to navigate housing and healthcare systems. Many described cycling in and out of housing and healthcare systems and waiting lists because the processes were difficult to navigate and sustain over time:

*I need support with important things*,* appointments*,* had a housing appointment this week*,* forgotten it and thought about it two days later. I went to (name of health service) for my diabetes. I was supposed to get to the clinic for a blood test. I got a reminder two days before*,* then they called me on the day. I told the woman “I can’t go. Can you reschedule me?” I get overwhelmed*,* confused about what is expected of me.* (Louise, region 1)

Another example from Denise was when trying to seek support from the social security system:

*I can’t keep going to the Newstart [social security payment; now known as JobSeeker Payment]. I keep forgetting. I can’t remember what you’re telling me. See I can’t. I tell them that. They say*,* “Write it down”. I say*,* “I lose the paper”. They tell me to save information in my phone or have a calendar. I am staying with my daughter.* (Denise, region 1)

Some frontline professionals, such as Deborah, felt crisis accommodation and temporary housing supports placed unrealistic expectations on Indigenous women who had experienced violence-related TBI to navigate various service systems:

*Sometimes I think there isn’t always the understanding or the workers in the shelters sometimes that has that very complex understanding of trauma and of brain injuries and almost expect someone coming into a shelter to just be independent and to be able to get*,* to be able to sort out the things going on without that extra support and if they do need that extra support or there’s some complications around that*,* then that then might limit their ability of how long they can stay in the shelter.* (Deborah, Housing and homeless service, region 2)

Another example shared by Luke related to managing multiple service system supports:

*I’ve worked with a number of clients who have said to me things like*,* “I’m very forgetful. I’ve had two knocks in the head. I got bashed as a little girl. I got bashed by my partner. I forget things really easily*,* so you have to remind me all the time. I can’t remember things. I need a chart”. So*,* the actual presentation when we’re working together is stuff like that really poor short-term memory and mixing up supports. Often people will think*,* I’ve had clients that go “I remember you gave me this thing” and it wasn’t me [housing and crisis support worker]*,* it was the health worker*,* but they mix the supports up.* (Luke, Housing and homeless service, region 2)

Frontline professionals reported that there was limited awareness and understanding of TBI within the crisis accommodation and housing service sector. In crisis accommodation settings, Indigenous women displaying TBI symptoms could be perceived as having ‘behavioural issues’ that were both disruptive for staff as well as other women staying at the shelter, or as being non-compliant with shelter rules, conditions and routines. Participants explained that this lack of awareness could add further layers of vulnerability for Indigenous women in housing systems. Difficulties associated with TBI - such as challenges with memory, communication, or emotional regulation - could affect a woman's ability to manage tenancy responsibilities. As a result, women could receive tenancy breaches or infractions recorded with the Department of Housing, which may have lasting consequences for their future access to stable housing:

*We’ve witnessed women that have obvious brain injuries because they’re not able*,* I guess*,* to control who’s coming and going and to have that ability to say*,* ‘No*,* you can’t be here’*,* then overcrowding and damage occurs and so*,* our public housing is actually evicting these really vulnerable women that have brain injuries that don’t have that capacity to understand what’s required of the tenancy or to say*,* ‘You can’t be here*,* you can’t*,* you have to stay out or you can’t be living here’ and so*,* then if they can’t secure housing with Department of Housing*,* it’s really difficult to accommodate these women*,* and I just think there needs to be more understanding from them around these particularly vulnerable cohort of women with brain injuries.* (Michael, community-based service, region 2)

## Discussion

This paper provides a detailed account on how the dynamics of different forms of homelessness, together with violence, interact to create barriers for Indigenous women seeking healthcare for TBI. Reflecting the impacts of imposed poverty, racism, discrimination, and settler-colonial patriarchy, prolonged and recurring experiences of homelessness feature within the lives of Indigenous women who have experienced family violence. Indigenous women experiencing violence often move between multiple forms of homelessness, including staying with family and friends, at crisis accommodation or hostels, and sleeping in public spaces [10]. For some Indigenous women in this study, particularly mothers, remaining or returning to an unsafe home may be the only option to avoid homelessness. Crisis accommodation is often perceived by Indigenous women as a place of safety [41]. However, services in regional and remote Australia are frequently stretched beyond capacity and unable to adequately meet local demand [3, 42]. The lack of affordable housing has long been a structural barrier, making private rental an unrealistic option for many Indigenous women attempting to leave violence.

TBI in the context of family violence has been described as ‘invisible’, reflecting both the limited awareness and understanding within the wider community and frontline service sectors and the fact that TBI can often occur without visible signs of injury to the head and neck [25, 26]. This study highlights how housing instability, alongside the urgent prioritization of safety and shelter, can further obsecure TBI generated by violence within the lives of women and across service systems. Due to the lack of immediate accommodation options in regional and remote areas, some Indigenous women in this study reported relocating from their home community or town to access crisis and short-term accommodation elsewhere. Such relocation can be particularly challenging given strong ties to extended family, Country and established local service networks [10]. Moving away may also undermine continuity of care, as Indigenous women could lose access to healthcare services in their home communities where trusting and respectful relationships with health professionals may already be estasblished [43, 44].

### Implications for policy and practice

The findings suggest several implications for housing services and systems in Australia. First, governments must adequately fund infrastructure and workforce capacity in regional and remote housing pathways (e.g., crisis, transitional and long-term social housing) to ensure accessible, affordable and secure housing for Indigenous women and their families. Such support can reduce the risk of exposure to violence and, in turn, the likelihood of violence-related TBI [45]. Safety and stable accommodation are the fundamental priorities for women who have experienced violence. Until these needs are met, the effectiveness of TBI care, support and treatment is likely to remain limited. Without access to appropriate accommodation, ongoing violence will continue to generate new, yet preventable forms of disability and complex health needs for Indigenous women and their children. Importantly, women, particularly Indigenous women and women living with disability, remain significantly underrepresented in governance and decision-making processes related to housing and funding decisions in settler colonial contexts [46]. Indigenous women hold critical knowledge and solutions to address violence in their lives; therefore, greater participation and leadership in decision-making, including control over how resources and funding are allocated, is essential to effectively respond to their housing needs [47].

Although not specifically focused on women with TBI from violence, there are several examples of rehabilitation initiatives and programs to support people experiencing homelessness to have access to healthcare, support and treatment following TBI [48–50]. Models that include on-site visits and the co-location of multidisciplinary teams - such as community health workers, social workers, occupational therapists and registered nurses - can strengthen the capacity of crisis accommodation and housing services to recognise and respond to TBI through the development of tailored protocols and practices. Screening for TBI is particularly important for underserved populations, as it enables the identification of cognitive and functional impacts and helps prioritise areas for treatment and support [48]. Health professionals can conduct screening and assessment outside traditional healthcare settings, allowing people experiencing homelessness to be assessed for cognitive and functional changes following TBI and to work collaboratively with practitioners to develop case plans that help manage symptoms and behavioural changes associated with TBI [48–50]. Multidisciplinary teams can also provide guidance on the physical design of services, including incorporation of low-stimulation environments and autonomy-supportive design principles that better accommodate the needs of people living with TBI [51, 52]. Further research is needed to examine the impact of multidisciplinary rehabilitation teams working in partnership with homelessness, crisis accommodation and housing services who support Indigenous women experiencing TBI. Such work could help develop evidence-based coordinated care pathways that better support the health, safety, and wellbeing of women and their children.

The need for increased education and training on TBI for both the community and frontline workforce is a well-documented recommendation in Australia and internationally [22, 24, 26, 45]. A further benefit of locating multidisciplinary health teams within crisis accommodation and housing services is their capacity to support both formal and informal TBI education and training for the housing workforce and people líving with TBI [48]. Common symptoms of TBI - such as aggression triggered by frustration, difficulties in communication or comprehension, fatigue and poor impulse control - can negatively affect people's ability to maintain housing and engage with support services following violence-related TBI [48]. When frontline workers are not provided with high-quality TBI education and its effects on everyday functioning, these symptoms may be misinterpreted as behavioural issues, such as acting out, lack of motivation, non-compliance with instructions, or disregard for service rules [22, 50]. Frontline service professionals in Australia have called for greater opportunities to develop the skills needed to hold safe and respectful conversations about TBI, facilitate appropriate referral pathways, and provide culturally appropriate supports that enable Indigenous women to manage TBI symptoms [7]. At the same time, limited awareness and education about TBI within the wider community can contribute to further stigma and isolation for people living with TBI [53]. Education initiatives must therefore be designed to recognise intersecting vulnerabilities related to race, racism, disability, gender, culture and socioeconomic inequality.

Finally, there are increasing calls for expanded services that support men to leave the home environment, alongside the development and delivery of men's healing programs and supports. Such approaches may reduce the need for Indigenous women to leave their communities or regions to access safe accommodation and healthcare. It also recognizes that men require connection with holistic services and supports in order to address and to end violence. Additional recommendations included having crisis accommodation services that do not exclude teenage boys [54]. 

### Strengths and limitations

This study involved Indigenous women from one regional and one remote location in Australia. While these regions are diverse and include Indigenous women who identify belonging to a range of different language and cultural groups, further insights into the intersecting dynamics of family violence, homelessness and access to health care for TBI could be gained from larger studies in the future. The project was guided by an advisory group that consisted of mostly Indigenous women working in service delivery or advocacy services, with the project methodology responsive to the guidance and knowledge shared by community Elders and community members [7]. Interviews with Indigenous women with lived experience of TBI from family violence were conducted through multiple interviews over several weeks to consider factors such as participant fatigue. However, these interviews were completed essenitally at one point in time. Longitudinal research that follows the journeys of Indigenous women from their point of first contact with services and across different housing pathways could further strengthen understanding of the dynamics of family violence, housing and healthcare access. Despite these limitations, the findings presented contribute to the literature by identifying the impacts of homelessness for Indigenous women living with TBI from family violence, highlighting both the opportunities and barriers related to accessing healthcare and support services, and providing important insights into the complex dynamics of homelessness and family violence.

## Conclusion

Safe and accessible housing is a fundamental right for all Indigenous women and their families. This study has identified how violence-related TBI remains unrecognized within housing systems that face longstanding structural barriers and have limited protocols to identify and respond to TBI. When TBI is not recognized or addressed, Indigenous women experiencing homelessness may face compounded vulnerability and ongoing exposure to violence. A well-resourced, integrated and responsive housing system for Indigenous women and their families has the potential to generate significant impacts - reducing repeated head injuries from violence, lowering the risk of violence-related disability, and contributing to longer-term cost savings for the health sector. These findings underscore the importance of policy and service initiatives that directly address the intersections of homelessness, family violence, healthcare, and disability through coordinated and collaborative responses. This is particularly critical given that Indigenous women experiencing TBI from family violence often encounter multiple barriers to access acute healthcare, including having injuries assessed and receiving appropriate referrals for ongoing care and support.

## Data Availability

No datasets were generated or analysed during the current study.
