# Correction: Exploring the dynamics between homelessness and healthcare access for Aboriginal and Torres Strait Islander women with traumatic brain injury from family violence: a qualitative study

**DOI:** 10.1186/s12889-026-29649-3

**Published:** 2026-10-07

**Authors:** Michelle Fitts, Karen Soldatic

**Affiliations:** 1 1Institute for Culture and Society, Western Sydney University, Parramatta, NSW Australia; 2 School of Health Research, Charles Darwin University, Alice Springs, NT Australia; 3 Institute of Tropical Health and Medicine, James Cook University, Townsville, QLD Australia; 4 Canada Excellence Research Chair, Toronto Metropolitan University, Toronto, ON Canada

**Correction: BMC Public Health 26**,** 2031 (2026)**

**https://doi.org/10.1186/s12889-026-26458-6**

During the process of publishing the original article [1], several textual and citation error were identified.

## Section Headings, Textual Corrections and Citation Renumbering

- “Unlike other terms, family violence reflects a wholistic understanding that includes violence that occurs within families and communities and, importantly, recognises broader aspects of the phenomenon including elder abuse, family feuding and child abuse.”

- Change to

- Unlike other terms, family violence reflects a holistic understanding that encompasses violence occurring within families and communities and, importantly, recognises broader aspects of the phenomenon, including elder abuse, family feuding, and child abuse.

- The section heading “Context of the study: the pathway from violence to homelessness for regional and remote Indigenous women in Australia” change to “Context of the study: the pathway from violence to homelessness for Indigenous women in regional and remote Australia”.
- TBI can occur even when a loss of consciousness is not experienced and when there no signs of injury (e.g., wound, bruising, blood) are visible.

- Change to:

- TBI can occur even when a loss of consciousness is not experienced and when there are no signs of injury visible (e.g., wound, bruising, blood).

- One crisis accommodation service had introduced a fee to stay in order to cover rising service delivery costs that could not be met due to limited funding.

- To this:

- One crisis accommodation service had introduced a small fee to help cover rising service delivery costs that could not be met due to limited funding.

- Alternative accommodation options offered to Indigenous women by family violence services included unsafe options such as tents.

- To this:

- Alternative accommodation options offered to Indigenous women by family violence services included unsafe **options such as tents**.

- Hostel rooms lacked adequate space for children’s belongings, such as clothing and toys, and facilities such as fridges were often too small to store items for larger families.

- To this:

- Hostel rooms often lacked sufficient space to store children’s belongings, such as clothing and toys, and facilities like fridges were frequently too small to store all items for large families.

- Reference citation under “Traumatic brain injury - the invisible disability generated by physical violence” reference 16 onwards were incorrectly renumbered.

The original article [1] has been corrected.
